# Bioconversion of Lactose into Glucose–Galactose Syrup by Two-Stage Enzymatic Hydrolysis

**DOI:** 10.3390/foods11030400

**Published:** 2022-01-30

**Authors:** Kristine Majore, Inga Ciprovica

**Affiliations:** Faculty of Food Technology, Latvia University of Life Sciences and Technologies, Rigas iela 22, LV-3004 Jelgava, Latvia; inga.ciprovica@llu.lv

**Keywords:** β-galactosidase, permeate, glucose isomerase, syrup, GOS

## Abstract

Fermentation technology enables the better use of resources and the conversion of dairy waste into valuable food products. The aim of this study is to evaluate the conversion rate of glucose into fructose by immobilised glucose isomerase (GI) in sweet and acid whey permeates for glucose–galactose syrup production. The experiments demonstrated that the highest concentration of glucose and galacto-oligosaccharides (GOSs) in sweet and acid whey permeates was reached by GODO-YNL2 β-galactosidase, 32 ± 2% and 28 ± 1%, respectively. After glucose isomerisation, the highest fructose yield was 23 ± 0.3% and 13 ± 0.4% in sweet and acid whey permeates, where Ha-Lactase 5200 β-galactosidase was used for lactose hydrolysis in sweet and acid whey permeates. Finally, the results of this study highlight the potential for two-stage enzymatic hydrolysis to increase the sweetness of glucose–galactose syrup made from sweet and acid whey permeates.

## 1. Introduction

Whey recycling has attracted a lot of interest in relation to lactose hydrolysis [1]. Sweet whey is widely used as the substrate for the production of whey protein concentrate, whey powder and lactose. On the other hand, due to high acidity and salt concentration and low protein concentration, there are still not so many technologies that can be diverted to acid whey processing [2].

β-Galactosidase has a meaningful role in the dairy industry, providing the conversion of lactose by its transferase and hydrolase activity into monosaccharides—glucose and galactose [3,4].

Fermentation is a significant step in production of glucose–galactose (GG) syrup. Glucose–galactose syrup is a viscous sugar solution consisting of about 30% water, 68% glucose and galactose, 11% lactose and 1% salt [5]. The syrup can be used as a sweet substance for ice cream, milk desserts, sauces and as an ingredient for caramel production. The sweetness of GG syrup is about 0.7 compared to sucrose [6]. β-Galactosidase usually catalyses the formation of 2–5 monomers of GOS, reaching up to 10 [7]. This can be explained by the fact that the glycosyl residue moves from the donor substrate to the acceptor molecule, creating a particular type of GOS [8]. The production of fructose from lactose includes two steps, i.e., first, lactose hydrolysis into glucose and galactose using β-galactosidase and, second, glucose isomerisation to fructose by glucose isomerase (GI) [9]. Fructose is usually used as a sweet substance in food and beverage production, as well as an additive for special food for diabetics [10]. It is sweeter than sucrose and can be absorbed more slowly than glucose, as well as being easily metabolised without the involvement of insulin [11].

Glucose isomerisation is a commonly used process, in the food industry, for the production of dietary products; it improves the sweetness of food and beverages, is capable of reducing unwanted colour pigments, has hygroscopic properties and increases viscosity [12]. Enzymatic isomerisation of glucose is used industrially in the United States, which is shown to be selective, requires low-energy consumption and develops less by-products and a better taste than chemical methods [13].

The aim of this study is to evaluate the conversion rate of glucose into fructose by glucose isomerase in sweet and acid whey permeates for glucose–galactose syrup production.

## 2. Materials and Methods

### 2.1. Chemicals and Materials

D-Lactose monohydrate (≥99.5% purity), D (+) galactose (≥99% purity), D (+) glucose (≥99.5% purity), KOH and acetonitrile were purchased from Sigma (St. Louis, MO, USA) and were of the highest quality available, unless otherwise stated.

β-Galactosidase (EC 3.2.1.23) NOLA Fit5500 (*Bacillus licheniformis* 5500 BLU g^−1^) is a soluble preparation from Chr. HANSEN (Hørsholm, Denmark), β-galactosidase is an Ha-Lactase 5200 (*Kluyveromyces lactis* 5200 NLU g^−1^) soluble preparation from Chr. HANSEN (Denmark) and GODO-YNL2 (*Kluyveromyces lactis* 5000 NLU g^−1^) is a soluble preparation from Danisco (Copenhagen, Denmark). Immobilised glucose isomerase (activity 350 U g^−1^) was isolated from *Streptomyces murinus* from Sigma-Aldrich (St. Louis, MO, USA).

Sweet and acid whey permeates with an initial solids’ concentration of 5% were donated by local dairy factories.

### 2.2. Analyses of Whey Permeate

The fat, protein, lactose and total solids’ concentration of permeate was determined by MilkoScan^TM^ Mars, Foss (Hillerød, Denmark). The macro-elements in permeate were measured by atomic absorption spectrophotometry (AAS) [14] and phosphate was measured by spectrophotometry [15].

### 2.3. Production of GG Syrup

#### 2.3.1. Hydrolysis of Permeate by β-Galactosidase

The whey solids’ concentration of 20% and enzyme load were selected based on the results found in previous studies [16,17]. The solids’ concentration was achieved using an evaporator (Armfield FT22; Ringwood, UK) under a vacuum at 0.56 bar. Three commercial β-galactosidases (0.05% each) were weighed and added to 100 mL of permeate. Substrate pH was controlled by 10% KOH. The samples were fermented in an incubator (Memmert IN55; Schwabach, Germany) at a temperature of 42.5 °C for 4 h. The experiments were carried out in triplicate.

#### 2.3.2. Isomerisation of Glucose

After 4 h of lactose hydrolysis, the hydrolysed permeate was used as a feedstock for glucose isomerisation, which contained a certain amount of glucose, galactose and left-over lactose. The pH of the permeate was adjusted to 7.5 by a 10% (100 g L^−1^) KOH solution; 1 g of glucose isomerase was added to each 100 mL of sample, which was then closed with aluminium foil to avoid vaporisation of the water. The samples were put into a thermostat at 70 °C for 12 h. After isomerisation, the samples were purified by filtration using a filter paper (cotton filter, pore size of 2.5 μm). Isomerisation conditions and parameters were performed according to Gaily et al. [18] with some modifications.

### 2.4. Chromatographic Determination of Carbohydrates

Carbohydrates (glucose, galactose, fructose and lactose) were analysed by high-performance liquid chromatography (HPLC). The samples were placed into 2 mL Eppendorf tubes and centrifuged for 5 min at 10,000 rpm. The samples were diluted to 1:10 with deionised water and 1 mL of the final sample was placed into a vial and sealed for the HPLC analysis (Shimadzu LC-20). Sugars were detected using a refractive index detector (RID) and SUPELCOSILTM LC-NH2 column 5 μm particle size, 25 cm × 4.6 mm. The temperature was set to 35 °C, the volume of the injected sample was 10 µL, the flow rate was 1.0 mL min^–1^ and the gradient mobile phase acetonitrile: deionised water (80:20) was used. The total analysis time was 15 min.

GOSs were determined by the HPLC method [19] using an Agilent 1100 chromatography system. The column used was a Shodex KS-802 (length 300 mm, ID 8 mm; refractive index detector; mobile phase—H_2_O; flow rate, 0.5 mL min^−1^). The total GOS concentration was determined and divided by the degree of polymerization, GOS2, GOS3 and GOS4. GOS2 was calculated by subtracting the lactose concentration from the total disaccharide concentration, determined by Shodex KS-802.

### 2.5. Data Analysis

The data were expressed as the mean ± standard deviation of three replicates. A comparison of the mean values was performed with a one-way analysis of variance using Tukey’s test (*p* < 0.05).

The degree of lactose hydrolysis (DLH) was expressed as the percentage of lactose conversion according to Equation (1).

(1) $$DHL=\left(\frac{1-RL}{IL}\right)\times 100$$

where, *RL*—lactose concentration (g L^−1^); *IL*—initial lactose concentration (g L^−1^).

Fructose and total GOS yields (Y) were expressed as a percentage according to Equation (2).

(2) $$Y=\left(\frac{\mathrm{obtained}\;\mathrm{sugar}\;\mathrm{amount}\;\left({g\;L}^{-1}\right)}{\mathrm{initial}\;\mathrm{lactose}\;\mathrm{concentration}\;\left({g\;L\;}^{-1}\right)}\right)\times 100$$

MS Excel 2019 software was used for all figures and statistical analyses.

## 3. Results and Discussion

### 3.1. Whey Permeate Composition

Comparing the data of sweet and acid whey permeates, a significant difference was established in the pH (Table 1).

It is important to point out that the lower the pH of the acid whey permeate, the higher the amount of soluble Ca^2+^ and PO_4_^3−^ which remains in the solution and cannot be separated by clarification. The cause of the low pH of acid whey permeate is the high content of organic acids, such as lactic and citric acids. Lactic acid formation from lactose fermentation occurs during the acid coagulation of casein [20].

Compared to acid whey permeate, the composition of sweet whey permeate varied with respect to lower concentrations of proteins and lactose as well as higher pH.

Table 2 demonstrates the amount of macro-elements and phosphate at different solids concentration (5% and 20%) in sweet and acid whey permeates.

All permeate samples contained a high amount of potassium. The largest difference in mineral composition was observed for Ca^2+^ and PO4^3−^ ions, the concentrations of which in acid whey permeate were approximately 3.5 and 2 times higher than those in sweet whey permeate.

Whey is considered a nutritionally valuable product because almost all macro- and micro-elements transfer into whey or whey permeate after milk coagulation [21,22]. Salts in milk can be found in the form of inorganic compounds, or as a part of proteins, fats and nucleic acids [23]. Several ions affect the activity of β-galactosidase [24]. The effect of ions on enzyme activity depends on the source of β-galactosidase and on the radius of ions, causing significant changes in enzyme behaviour [25]. The combination of sodium and potassium with chloride gives salinity, demonstrating that, during the production of GG syrup from acid whey permeate, the syrup has a salty taste [26,27]. In addition, acid whey has a higher lactic acid concentration and lower pH than sweet whey, which is a major factor that strongly influences β-galactosidase activity and the ability to hydrolyse lactose in acid whey. Each enzyme has an optimum pH for the hydrolysis reaction and, if it is necessary, to raise the pH of the substrate, as a recommendation is to add alkali. Therefore, it immediately changes the ion concentration and enzyme activity in the substrate [28].

### 3.2. Enzymatic Hydrolysis and Transgalactosylation in Whey Permeate

The first stage to produce GG syrup from lactose is the enzymatic hydrolysis of lactose.

**Figure 1:**
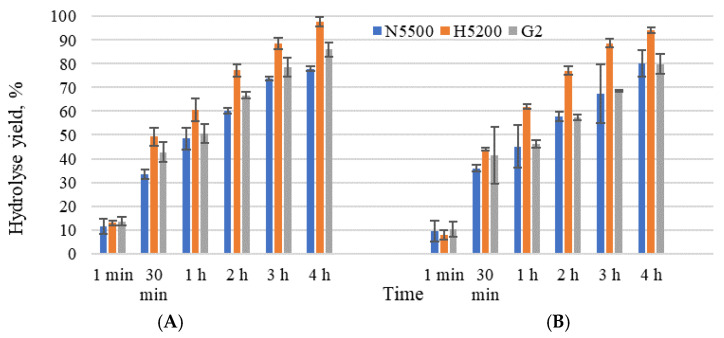
Hydrolysis yield (%) with studied β-galactosidases. Sweet whey permeate solids of 20% (**A**) and acid whey permeate solids of 20% (**B**). G2—GODO-YNL2; N5500—NOLA™Fit5500; H5200—Ha-Lactase 5200. Mean ± standard deviation; *n* = 3.

The initial lactose concentrations of sweet whey permeate (205 ± 3 gL^−1^) and acid whey permeate (180 ± 10 gL^−1^) were hydrolysed within 4 h, achieving hydrolysis yield in the range 78–97%. A similar kinetic trend was observed with GODO-YNL2 and NOLA™Fit5500 β-galactosidase (Figure 1A,B) during 4 h of lactose hydrolysis, with a hydrolysis yield of 86% and 78% using sweet whey permeate, but 80% for both using acid whey permeate. It was challenging to achieve at least an 80% hydrolysis yield using β-galactosidase, due to the formation of galactose and glucose in the presence of permeate salts, acids and proteins. Czyzewska et al. [29] confirmed that the presence of glucose in the hydrolysed substrate strongly affects the activity of NOLA™ Fit5500 β-galactosidase. Inhibition slows the rate of hydrolysis and decreases the enzymatic activity, which, in turn, prolongs the hydrolysis reaction [30]. The samples with Ha-Lactase 5200 β-galactosidase demonstrated that, after 4 h, the hydrolysis yield achieved was 97.9 ± 0.1% and 94.1 ± 1.0% in the sweet whey and acid whey permeates, respectively.

The concentration and profile of carbohydrates change over time, varying considerably depending on the type of permeate and the concentration of salts [31].

Lactose hydrolysis in sweet whey permeate resulted in a higher concentration of monosaccharides than in the acid whey permeate samples. After 4 h of lactose hydrolysis using sweet whey permeate, the obtained glucose was in an approximate range from 36 to 51% and galactose from 15 to 21% of the total sugars, but, using acid whey permeate, these were from 32 to 51% and from 17 to 25% of the total sugars. After a period of time, the equimolar concentration in the reaction of glucose and galactose differed; using sweet whey permeate, the glucose concentration was higher than galactose starting from the first minute of hydrolysis, but, for the acid whey permeate samples, only after 30 min.

The conversion factor for lactose forming into glucose and galactose ranged from 1.05 to 1.11 [32], which means that the β-galactosidase started to produce galacto-oligosaccharides under certain conditions, such as water activity, galactose concentration and enzyme origin [33]. This activity begins when the water activity becomes more optimal, near or above 0.6 for the β-galactosidase side reactions, and the sugar concentration changes; the galactose concentration decreases by the transgalactosylation reaction in concentrated lactose solutions [34,35].

**Figure 2:**
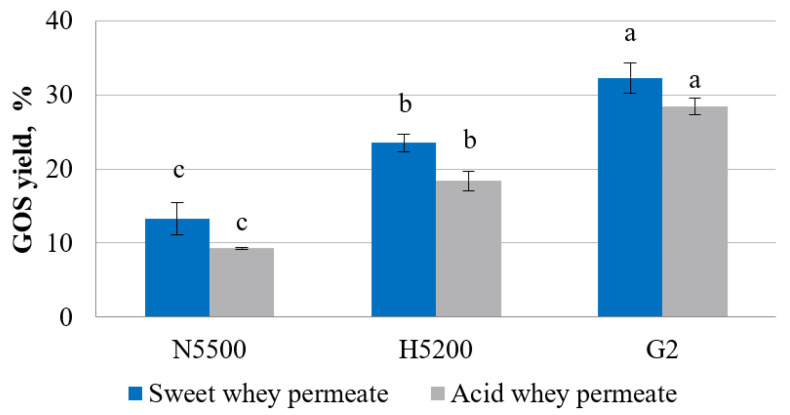
GOS yield in sweet and acid whey permeates with solids of 20% after lactose hydrolysis. G2—GODO-YNL2; N5500—NOLA™ Fit5500; H5200—Ha-Lactase 5200. The values marked with the same letter within each enzyme did not differ significantly (*p* > 0.05). Mean ± standard deviation; *n* = 3.

The yield of GOS, Figure 2, which was noticeably lower in hydrolysed acid whey permeate, was in the range from 9 ± 0.2 to 28 ± 1%, but, in sweet whey permeate, it was from 13 ± 2 to 32 ± 2% of the total sugars. The experiment showed that the highest production activity of GOSs was shown by GODO-YNL2 β-galactosidase, but the lowest activity was observed with NOLA^TM^ Fit5500 β-galactosidase. The factors which impact the yield of GOSs are temperature, pH, reaction time, source of β-galactosidase and the initial concentration of lactose [36]. Luzzi et al. [34] hydrolysed lactose using four commercial β-galactosidases where the lowest GOS yield of 71 g L^−1^ was obtained by NOLA^TM^ Fit5500 β-galactosidase. In turn, a similar study was carried out by Venica et al. [37], where GODO-YNL2 β-galactosidase was used for the production of GOSs and the researchers found that using a lactose concentration of 20 g per 100 mL, the maximal GOS yield of 26% could be obtained. The origin of β-galactosidase is an important factor in determining the GOS yield in the samples; the enzyme from *Kluyveromyces lactis* produces β-(1→6) galacto-oligosaccharides, mainly 6′-galactosyllactose, allolactose and 1-6-β-D-galactobiose, but, from *Bifidobacterium bifidus*, GOSs are primarily produced with a β-(1→3) glycosidic bond [38].

**Figure 3:**
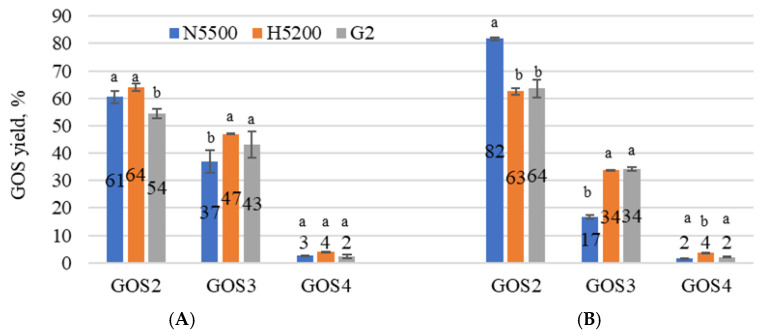
Types of GOSs in sweet whey permeate with solids of 20% (**A**) and in acid whey permeate with solids of 20% (**B**) samples after lactose hydrolysis. G2—GODO-YNL2; N5500—NOLA™ Fit5500; H5200—Ha-Lactase 5200. The values marked with the same letter within each GOS did not differ significantly (*p* > 0.05). The number on the bar indicates the concentration (g L^−1^) of GOSs in the sample. Mean ± standard deviation; *n* = 3.

To determine the most productive β-galactosidase for GOS synthesis in sweet and acid whey permeates, each GOS form was expressed as a percentage of the total amount of GOSs in the sample. Each enzyme showed a specific transgalactolytic activity, see Figure 3A,B. Disaccharides (GOS2) that could be produced by β-galactosidases were galactobiose, allolactose, trisaccharide (GOS3)—6′ galactosyl lactose and a small concentration of tetrasaccharides (GOS4) could also be detected. The highest GOS2 production was achieved by NOLA^TM^ Fit5500 β-galactosidase, 84 ± 1% in acid whey permeate, and that of GOS3 by GODO-YNL2 and Ha-Lactase 5200 β-galactosidases, 47 ± 0.1 and 43 ± 4% in sweet whey permeate, respectively. In both permeates, the enzymes showed a tendency to produce a large amount of GOS2. Allolactose is a disaccharide, a lactose isomer, where, instead of the β-1→6 glycosidic bond, there is a β-1→4 bond. This indicates that β-galactosidase plays an additional role in bond modification [39]. Nutritional studies have shown combinations of GOSs with different chain lengths to maximize fermentative and prebiotic effects [40]. The transgalactosylation behaviour of β-galactosidases strongly depends on the concentration of salts in whey and their ratio [41]. It should be highlighted that the reaction of hydrolysis and transgalactosylation could also be influenced by the addition of a 10% KOH solution, which was used to adjust the pH of the substrate, thus increasing the K^+^ concentration, as well as activating enzyme activity.

These results indicate the ability of each β-galactosidase to form GOSs with different structures and the ability to produce GOSs with potentially different prebiotic properties in GG syrup.

### 3.3. Effect of Two-Stage Enzymatic Hydrolysis to Increase Syrup Sweetness

**Figure 4:**
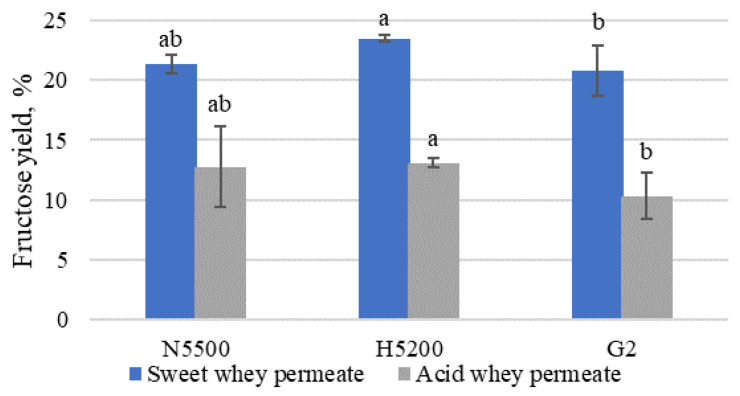
Fructose yield after glucose isomerisation of hydrolysed permeate. G2—GODO-YNL2; N5500—NOLA™ Fit5500; H5200—Ha-Lactase 5200. The values marked with the same letter within each enzyme did not differ significantly (*p* > 0.05). Mean ± standard deviation; *n* = 3.

The fructose yield in Figure 4 was observed to be higher in sweet whey permeate than in acid whey permeate.

The highest fructose yieldwas shown in samples where Ha-Lactase 5200 β-galactosidase was used for lactose hydrolysis. The fructose yield of 29.45 ± 2.35% was reported by Cheng et al. [42], 45.3% was reported by Wang et al. [13] and 52.16% was reported by Jia et al. [43].

One of the factors influencing the formation of fructose in acid whey permeate is metal ions. Li et al. [44] reported that divalent metal ions inhibited glucose isomerase activity, even at a small concentration of Ca^2+^. Table 1 demonstrates that Ca^2+^ concentration in both permeates varied from 1071 ± 99 mg kg^−1^ to 3400 ± 86 mg kg^−1^.

On the other hand, the reaction conditions are very important for the formation of fructose. Under certain conditions—pH and temperature—fructose can be converted back to glucose [11], which means that the enzymatic isomerisation of glucose–fructose is reversible, based on the literature review. This indicates that glucose isomerase is sensitive and can be easily affected.

**Figure 5:**
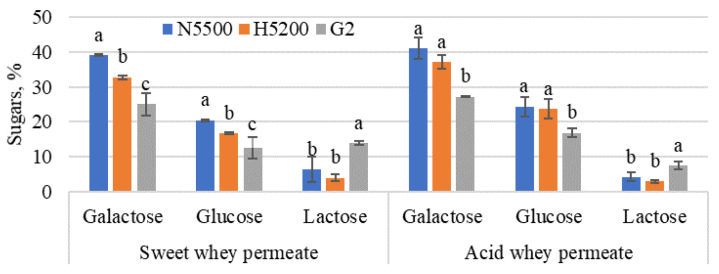
The overall yield of sugars was derived from the enzymatic hydrolysis of lactose, followed by glucose isomerisation. G2—GODO-YNL2; N5500—NOLA™ Fit5500; H5200—Ha-Lactase 5200. The values marked with the same letter within each sugar did not differ significantly (*p* > 0.05). Mean ± standard deviation; *n* = 3.

After hydrolysis, the glucose concentration was higher than galactose, but, after isomerisation (see Figure 5), the result was the opposite. The galactose and glucose in sweet whey permeate varied from 25 ± 3.2 to 39 ± 0.2% and from 13 ± 3.1 to 20 ± 0.3% of the total sugars, respectively, but, in acid whey permeate, they varied from 27 ± 0.1 to 41 ± 3.0% and from 17 ± 1.3 to 24 ± 2.8% of the total sugars, respectively. In turn, the residual lactose yield in sweet whey permeate was from 4 ± 1 to 14 ± 0.5% and, in acid whey permeate, from 3 ± 0.4 to 7 ± 1.0% of the total sugars. Comparing the amount of glucose and galactose after 4 h of lactose hydrolysis (Table 3) and after glucose isomerisation (Figure 5), there were significant differences. β-Galactosidase was not completely inactivated; for example, the NOLA^TM^ Fit5500 enzyme had a high possibility of being active for a while; the pH and temperature of permeate changed immediately after 4 h of hydrolysis. β-Galactosidase had time to continue the reaction while the pH and temperature changed. The addition of 10% KOH raised the permeate pH to 7.5, which played an important role in enzyme productivity. Foda et al. [45] reported that monovalent ions such as Na^+^ and K^+^ acted as activators of glucose isomerase. Two-stage hydrolysis made it possible to produce syrup containing fructose in the range 10–20%. This greatly improved the intensity of the sweetness of GG syrup and increased its potential to be used as a sugar substitute.

The results of this study will be used to assess the perspective of two-stage enzymatic hydrolysis and the importance of using it to enhance the nutritional value and potential health benefits of glucose–galactose syrup.

## 4. Conclusions

This work shows that whey could be transformed into a valuable food product using a green recycling method. Two-stage hydrolysis of permeate significantly increased the sweetness and overall sugar composition of the GG syrup. A few studies have used acid whey for two-stage hydrolysis. Our research study provides useful data for other scientists to compare and draw conclusions in the future research. Comparing the amount of fructose between permeates, a higher fructose yield was obtained using sweet whey permeate. Substrate composition and reaction conditions are very important for fructose formation, because glucose isomerase is sensitive and susceptible to reaction conditions (pH, temperature, etc.). We show that all enzymes can produce more GOSs in sweet whey permeate and GODO-YNL2 had the highest GOS-formation activity. The origin of β-galactosidase plays an important role in the production of GOS because it affects the reaction conditions and reaction performance.

## Figures and Tables

**Table 1:** Composition of sweet and acid whey permeates used in the study and pH. Mean ± standard deviation; *n* = 3.

Permeates	Fat (%)	Proteins (%)	Lactose (%)	Total Solids (%)	pH
Sweet whey, 5%	<0.1	0.2 ± 0.1	3.8 ± 0.1	4.6 ± 0.1	6.1 ± 0.1
Acid whey, 5%	<0.1	0.5 ± 0.1	4.2 ± 0.2	5.2 ± 0.2	4.6 ± 0.1
Sweet whey, 20%	<0.1	0.7 ± 0.1	18.8 ± 0.1	20.7 ± 0.2	6.2 ± 0.1
Acid whey, 20%	<0.1	1.2 ± 0.2	18.2 ± 0.2	20.3 ± 0.3	4.5 ± 0.1

**Table 2:** Concentration (mg kg^−1^) of macro-elements and phosphate in different solid permeates. Mean ± standard deviation; *n* = 3.

Permeates	Ca^2+^	Na^+^	K^+^	Mg^2+^	PO_4_^3−^
Sweet whey, 5%	349 ± 14	429 ± 11	1480 ± 100	57 ± 9	275 ± 61
Acid whey, 5%	1322 ± 83	469 ± 67	1636 ± 189	123 ± 16	736 ± 34
Sweet whey, 20%	1071 ± 99	660 ± 57	2710 ± 55	260 ± 13	1265 ± 50
Acid whey, 20%	3400 ± 86	1100 ± 55	5600 ± 140	340 ± 18	2200 ± 56

**Table 3:** Number of sugars (%) after lactose hydrolysis using different permeates with solids of 20%. Mean ± standard deviation; *n* = 3.

		Time					
Enzyme	Sugar	1 min	30 min	1 h	2 h	3 h	4 h
Sweet whey permeate							
N5500	Glucose	2.6 ± 0.2 ^c^	8.1 ± 0.4 ^e^	29.9 ± 0.1 ^a^	33.0 ± 1.0 ^c^	41.9 ± 0.2 ^b^	43.9 ± 1.4 ^b^
	Galactose	1.6 ± 0.1 ^b^	3.8 ± 0.2 ^e^	12.4 ± 0.5 ^c^	14.9 ± 0.7 ^b^	15.5 ± 1.0 ^c^	16.4 ± 0.9 ^d^
	Lactose	88.6 ± 1.1 ^bc^	66.6 ± 0.1 ^a^	51.5 ± 0.6 ^c^	39.8 ± 1.2 ^b^	26.3 ± 1.1 ^b^	22.1 ± 0.5 ^a^
H5200	Glucose	3.1 ± 0.1 ^b^	21.9 ± 0.5 ^a^	30.0 ± 0.4 ^a^	43.9 ± 1.5 ^a^	46.5 ± 2.9 ^a^	51.0 ± 3.4 ^a^
	Galactose	1.6 ± 0.1 ^b^	8.3 ± 0.6 ^c^	11.7 ± 0.4 ^c^	16.8 ± 0.3 ^a^	17.0 ± 2.5 ^b^	20.9 ± 1.1 ^b^
	Lactose	87.1 ± 0.7 ^c^	50.8 ± 0.9 ^d^	39.5 ± 0.1 ^e^	22.7 ± 0.9 ^d^	11.4 ± 1.2 ^d^	2.1 ± 0.6 ^e^
G2	Glucose	1.9 ± 0.1 ^d^	13.9 ± 1.0 ^c^	25.0 ± 0.6 ^b^	29.7 ± 1.6 ^d^	35.9 ± 0.6 ^d^	36.0 ± 0.3 ^c^
	Galactose	0.9 ± 0.1 ^c^	6.5 ± 0.1 ^d^	11.2 ± 1.2 ^cd^	12.8 ± 1.7 ^c^	14.9 ± 1.5 ^c^	15.2 ± 0.1 ^e^
	Lactose	86.5 ± 1.1 ^c^	57.2 ± 1.2 ^c^	49.4 ± 0.7 ^d^	33.2 ± 0.9 ^c^	21.4 ± 0.1 ^c^	13.9 ± 0.6 ^c^
Acid whey permeate							
N5500	Glucose	7.1 ± 0.5 ^a^	10.8 ± 1.3 ^d^	15.9 ± 1.9 ^c^	26.7 ± 2.6 ^d^	40.8 ± 2.4 ^b^	43.5 ± 1.6 ^b^
	Galactose	7.7 ± 1.6 ^a^	10.5 ± 0.6 ^b^	14.0 ± 0.5 ^b^	15.2 ± 1.2 ^b^	21.0 ± 0.5 ^a^	25.3 ± 1.7 ^a^
	Lactose	90.5 ± 0.8 ^ab^	64.0 ± 1.0 ^b^	54.9 ± 0.3 ^a^	42.3 ± 1.3 ^ab^	32.7 ± 0.8 ^a^	19.8 ± 0.5 ^b^
H5200	Glucose	5.7 ± 1.2 ^a^	14.7 ± 0.7 ^c^	23.6 ± 0.5 ^a^	38.0 ± 1.8 ^b^	47.8 ± 1.3 ^a^	50.6 ± 2.1 ^a^
	Galactose	6.2 ± 1.8 ^a^	11.0 ± 0.6 ^b^	15.4 ± 2.2 ^c^	16.8 ± 1.3 ^ab^	17.6 ± 0.6 ^b^	22.4 ± 1.4 ^ab^
	Lactose	92.2 ± 1.1 ^a^	56.1 ± 1.3 ^c^	38.1 ± 0.8 ^e^	22.8 ± 0.9 ^d^	11.3 ± 1.0 ^d^	5.9 ± 0.3 ^d^
G2	Glucose	6.0 ± 1.0 ^a^	16.0 ± 0.3 ^b^	16.3 ± 1.1 ^c^	22.5 ± 1.7 ^e^	26.9 ± 1.3 ^c^	32.5 ± 1.7 ^d^
	Galactose	6.8 ± 1.8 ^a^	13.0 ± 0.2 ^a^	10.3 ± 0.1 ^d^	12.7 ± 0.4 ^c^	15.4 ± 1.6 ^b^	17.2 ± 1.6 ^c^
	Lactose	89.8 ± 1.1 ^b^	58.6 ± 1.3 ^c^	53.7 ± 0.3 ^b^	42.4 ± 0.8 ^a^	31.5 ± 1.3 ^a^	20.2 ± 1.0 ^b^

G2—GODO-YNL2; N5500—NOLA™ Fit5500; H5200—Ha-Lactase 5200. The values with the same letter within each sugar did not differ significantly (*p* > 0.05).

## Data Availability

The data in this study are available upon request.
